# Pleistocene Niche Stability and Lineage Diversification in the Subtropical Spider *Araneus omnicolor* (Araneidae)

**DOI:** 10.1371/journal.pone.0121543

**Published:** 2015-04-09

**Authors:** Elen A. Peres, Thadeu Sobral-Souza, Manolo F. Perez, Isabel A. S. Bonatelli, Daniel P. Silva, Márcio J. Silva, Vera N. Solferini

**Affiliations:** 1 Department of Genetics, Evolution and Bioagents, Institute of Biology, University of Campinas, Campinas, São Paulo, Brazil; 2 Department of Biology, Federal University of São Carlos, Sorocaba, São Paulo, Brazil; 3 Instituto de Ciências Biológicas, Universidade Federal do Pará, Belém, Pará, Brazil; 4 Center for Molecular Biology and Genetic Engineering, University of Campinas, Campinas, São Paulo, Brazil; University of Innsbruck, AUSTRIA

## Abstract

The influence of Quaternary climate oscillations on the diversification of the South American fauna is being increasingly explored. However, most of these studies have focused on taxa that are endemic to tropical environments, and relatively few have treated organisms restricted to subtropical biomes. Here we used an integrative phylogeographical framework to investigate the effects of these climate events on the ecological niche and genetic patterns of the subtropical orb-weaver spider *Araneus omnicolor* (Araneidae). We analyzed the mitochondrial (Cytochrome Oxidase I, COI) and nuclear (Internal Transcribed Subunit II, ITS2) DNA of 130 individuals throughout the species’ range, and generated distribution models in three different climate scenarios [present, Last Glacial Maximum (LGM), and Last Interglacial Maximum (LIG)]. Additionally, we used an Approximate Bayesian Computation (ABC) approach to compare possible demographic scenarios and select the hypothesis that better explains the genetic patterns of *A*. *omnicolor*. We obtained high haplotype diversity but low nucleotide variation among sequences. The population structure and demographic analyses showed discrepancies between markers, suggesting male-biased dispersal in the species. The time-calibrated COI phylogenetic inference showed a recent diversification of lineages (Middle/Late Pleistocene), while the paleoclimate modeling indicated niche stability since ~120 Kya. The ABC results agreed with the niche models, supporting a panmictic population as the most likely historical scenario for the species. These results indicate that *A*. *omnicolor* experienced no niche or population reductions during the Late Pleistocene, despite the intense landscape modifications that occurred in the subtropical region, and that other factors beside LGM and LIG climate oscillations might have contributed to the demographic history of this species. This pattern may be related to the high dispersal ability and wide environmental tolerance of *A*. *omnicolor*, highlighting the need for more phylogeographical studies with invertebrates and other generalist taxa, in order to understand the effects of Quaternary climate changes on Neotropical biodiversity.

## Introduction

Quaternary climate oscillations are recognized as important drivers of speciation and lineage diversification in many taxa, and have been intensely investigated through phylogeographical approaches in recent decades. The genetic and demographic consequences of these events are well documented for the Northern Hemisphere [1,2]; nevertheless, many megadiverse areas in the Southern Hemisphere are still little studied [1,3,4].

In the Neotropical region, the Quaternary Period is characterized by cycles of drier and wetter conditions that caused drastic spatial rearrangements of forest and savanna biomes [5–9]. According to some authors, these landscape modifications potentially induced population’ retractions and expansions in many species, which would explain the intense lineage diversification attributed to this period (the refuge theory) [10,11]. However, this issue is still controversial and highly debated, since paleoecological studies contradict the refuge hypothesis [12,13], and divergence times in several Neotropical groups indicate that biodiversity in the Neotropics might also be shaped by Tertiary orogenic events [3,9,14].

Despite the recent increase of phylogeographic studies in South America, there is still a large discrepancy among the biomes and regions analyzed. Almost half of the surveys (47%) have focused only on specific biomes [3], limiting the investigation to endemic groups. Furthermore, environments other than tropical forests are still little studied: the subtropical portion of the continent, for example, was treated in only ca. 6% of these surveys [3].

In Brazil, this subtropical region has mean annual temperatures between 10 and 15°C and short or no dry periods during the year. The landscape is a mosaic of phytophysiognomies, including the Atlantic Rainforest, semideciduous forests, *Araucaria* woodlands (with predominance of the Brazilian pine *Araucaria angustifolia*), savannas and grasslands [5,15]. Palaeoecological studies and pollen records suggest that the floristic composition of South American subtropical region underwent drastic changes during the Late Pleistocene: the climate in the LGM was drier and 5 to 7°C cooler and the grasslands, found today only in highland patches in southern Brazil, expanded more than 750 km northward and became the predominant biome of the region in this period [5,6]. However, the impact of these changes on the subtropical fauna is controversial, and the responses of individual species to these events varied substantially [16–20].

In recent years, the use of phylogeography to study the effects of past climate changes on biodiversity has been aided by new tools that provide *a priori* hypotheses to be tested, overcoming the major limitations in this field [21–24]. Species distribution modeling, for example, allows population geneticists to generate demographic hypotheses based on the distribution of organisms in current and past climate conditions. In addition to these geospatial methods, model-based approaches, such as the Approximate Bayesian Computation (ABC) [25–27], have been increasingly employed, allowing statistical comparison among alternative complex demographic scenarios with fewer computational limitations than likelihood-based methodologies.

Given the lack of surveys that focus on the influence of Quaternary climate oscillations on the diversification of the subtropical South American fauna (especially for invertebrates), here we integrated phylogeographic analyses of mitochondrial and nuclear DNA with geospatial and model-based methods to investigate the demographic history of the subtropical orb-weaver spider *Araneus omnicolor* (Araneidae). These spiders are commonly found in southern Atlantic Forest fragments, but also in drier environments and secondary/mixed forests in agroecosystems (i.e., not endemic to a specific biome, personal observations). The narrow subtropical occurrence of *A*. *omnicolor* suggests that climate might have influenced the establishment and evolutionary history of the species. However, so far, no studies have addressed arachnid demographic histories in this environment.

To evaluate the influence of Pleistocene climate events on the species’ ecological niche and genetic patterns, we generated distribution models in three different climate scenarios [present, Last Glacial Maximum (LGM, ~21 Kya) and Last Interglacial Maximum (LIG, ~120 Kya)]. Additionally, we used model-based ABC analyses to compare alternative demographic scenarios and select the model that better explains the empirical genetic data. We hypothesized that if these climate oscillations had affected *A*. *omnicolor* and reduced the populations to isolated patches, we would observe a strong population structure and demographic bottlenecks associated with niche reduction and/or fragmentation. This is the first survey to apply an integrative framework to explore the effects of past climate events on the phylogeographic patterns of a subtropical taxon in South America.

## Materials and Methods

### Sample collection and DNA extraction

**Fig 1:**
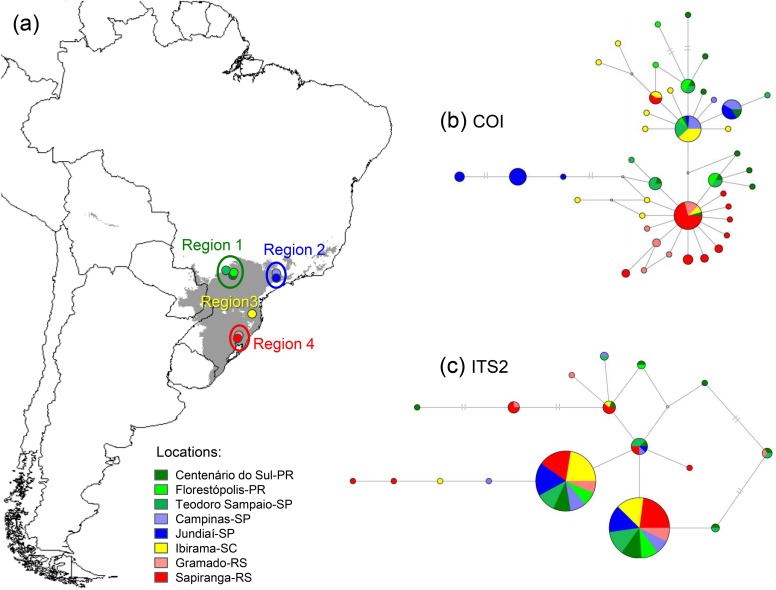
Study region and median-joining haplotype networks. (a) Sampling locations of *Araneus omnicolor* (separated by geographical regions). The current species distribution area calculated by climate models is shown in gray. (b) Mitochondrial and (c) nuclear networks. Circle sizes represent haplotype frequencies; colors correspond to sample locations on the map.

We sampled 130 individuals of *A*. *omnicolor* from eight locations (separated into four geographical regions, because of the proximity of some populations), covering most of the species distribution (Fig. 1A, S1 Fig.). Genomic DNA was extracted from legs with the Wizard Genomic DNA Purification kit (Promega), according to the manufacturer’s protocol. The abdomens were used for species confirmation, and the vouchers were catalogued in the Coleção Científica de Aracnídeos e Miriápodes of the Instituto Butantan. All specimens were collected under permits granted by the Instituto Chico Mendes de Conservação da Biodiversidade (ICMBio, permit nos. 14147, 22645, 32664).

### DNA amplification and sequencing

The Cytochrome Oxidase I (COI) region was amplified in the samples with primers LCO1490 and HCO2198 [28] under the following conditions: an initial denaturation step at 94°C for 3 min; 35 cycles at 94°C for 45 s, 55°C for 45 s and 72°C for 2 min; and a final extension step at 72°C for 3 min. The Internal Transcribed Subunit II (ITS2) nuclear region was amplified using primers 5.8S and 28S [29] under similar conditions: 95°C for 4 min; 35 cycles at 95°C for 45 s, 62°C for 45 s and 72°C for 2 min; and extension at 72°C for 10 min. The amplicons were analyzed in a Perkin-Elmer Prism 377 capillary sequencer. Sequences were aligned using the MUSCLE algorithm [30] and manually inspected and edited in MEGA 6.0 [31]. We first coded heterozygous sites in ITS2 sequences according to IUPAC ambiguity codes. Individuals with alleles containing indels had their heterozygous positions resolved with the method described by Flot *et al*. [32] in the software Champuru 1.0 [33].

### Haplotype reconstruction, genetic diversity and population structure

Median-joining haplotype networks were obtained using the software NETWORK 4.611 [34]. Phased ITS2 haplotypes were previously estimated using a Bayesian method implemented in PHASE [35] based on the input files prepared with SeqPHASE [36]. The gametic phases were inferred with a minimum posterior probability of 0.6, a level that has been suggested as optimal for reducing the number of unresolved haplotypes with fewer false positives [37]. Signs of past recombination were tested using a PHI Test [38] in the software SplitsTree4 [39].

We calculated haplotype (h) and nucleotide (π) diversity and the number of polymorphic sites (S) in each population, each geographical region, and in the total dataset in DnaSP v5.10 [40]. Genetic distances between populations (corrected mean number of nucleotide substitutions between populations, *D*_A_ [41]) were estimated in ARLEQUIN 3.5 [42]. To assess population structure, we calculated pairwise Φ_ST_ values and conducted an analysis of molecular variance (AMOVA [43]) in ARLEQUIN 3.5 [42] to determine the hierarchy of the genetic structure among the geographical regions. The statistical correlations between geographic distances and population differentiation (genetic distances and pairwise Φ_ST_) were investigated through Mantel tests [44].

### Phylogenetic inferences and divergence times

Bayesian inference trees were constructed in BEAST 1.7.4 [45] for COI and ITS2 datasets separately (the combined multilocus analysis was impossible due to the large number of ITS2 heterozygotes with different lengths). The models of nucleotide substitution that best fit our data (HKY+G and HKY for COI and ITS2, respectively) were previously selected using the AIC criterion in jMODELTEST 0.1.1 [46], and an outgroup (*Araneus venatrix*) was included to root the trees.

We applied a lognormal relaxed clock for the COI dataset (selected through a Bayes Factor analysis as the most appropriate evolution model: log_*e* lognormal clock_—log_*e* strict clock_ = 7.72 [47,48]) and a strict clock for ITS2. Given the scarcity of fossil records for the group, we used the COI substitution rate of 0.0115 per million years to calibrate the nodes in the COI tree and estimate the divergence times. This rate was proposed for insects by Brower [49] and is widely used in phylogenetic and phylogeographic studies with araneomorph spiders [50–53]. We carried out two independent runs of 200 million generations each, and sampled trees every 4000 generations. We checked for the convergence to a stationary distribution and for high effective sample sizes (ESS>200) in Tracer 1.5 [54]. The first 5000 trees were discarded as burn-in in TreeAnnotator, and the resulting trees were drawn in Figtree 1.4 [55].

### Demographic analyses

We used neutrality tests (Tajima’s D [56] and Fu’s Fs [57]) to infer historical demographic processes (i.e., recent expansions, bottlenecks, selection, etc.) in each population, each geographical region, and in the whole dataset in ARLEQUIN 3.5 [42]. We also conducted mismatch distribution analyses [58,59] for each region and for the total of sequences in ARLEQUIN 3.5, to determine the distribution of frequencies of pairwise differences. In these analyses, unimodal curves and non-significant values of the raggedness index (*r*) indicate that populations do not deviate from the expected model of rapid expansion.

Finally, we performed a multilocus Extended Bayesian Skyline Plot (EBSP) analysis in BEAST 1.7.4 [45] to infer changes in the effective population size through time [60]. We unlinked substitution, clock and tree models of the two loci, and specified a linear model of population size, instead of the less-realistic stepwise model. As in the phylogenetic inferences, we applied the most suitable nucleotide substitution models (HKY+G and HKY for COI and ITS2, respectively) selected in jMODELTEST 0.1.1 [46] and the COI mutation rate of 0.0115/My for node calibration. The weights for EBSP operators and the initial value for the mean population size were adjusted to improve MCMC mixing, according to the recommendations of the tutorial in the BEAST website. For each simulation two independent runs were performed, with 200 million generations each and samples taken every 10 000 generations. We used Tracer v1.5 [54] to check the quality of the parameters, and generated an annotated tree for each region with TreeAnnotator, discarding the first 2000 trees. The final plot was based on the output produced by the combined results.

### Species distribution and paleoclimate modeling

We compiled occurrence records for *A*. *omnicolor* from different sources to generate the species distribution models: a taxonomic revision for the genus [61], zoological collections [Coleção Científica de Aracnídeos e Miriápodes of the Instituto Butantã, Coleção de Aracnídeos of the Museu de Ciências e Tecnologia da PUCRS (MCTP), Museu de Zoologia of the Universidade Estadual de Campinas and Sistema de Informação Ambiental do Programa Biota/Fapesp-SinBiota] and new records from our own sample collections. A total of 52 unique occurrence points were obtained, considering the grid cell resolution used (S1 Fig.).

To test the effects of past climate oscillations on the species’ niche, we fitted the models in current, 21 Kya (LGM), and 120 Kya (LIG) scenarios using three different algorithms (applying the default settings of each package) to minimize possible biases: GARP with best subsets [62] and Support Vector Machines [63,64] (SVM hereafter), as implemented in OpenModeller [65]; and the Maximum Entropy algorithm, as implemented in Maxent 3.3.3 [66,67]. These algorithms are based on artificial intelligence methods and correctly predict the known distribution of species more often than other, simpler procedures [68].

The bioclimate variables used in the modeling approaches were chosen from the 19 layers available in the WordClim dataset (http://www.worldclim.com) through a jackknife procedure using Maxent, a method that minimizes over-parametrization issues and allows the algorithms to produce biologically more reliable distributions [67,69]. The five variables with the highest contributions to the analysis were selected (precipitation of driest month, maximum temperature of warmest month, precipitation of warmest quarter, mean temperature of coldest quarter, and temperature seasonality), except for the ‘Mean Diurnal Range’, which was not included because of its low permutation importance value (S1 Table). The bioclimate layers were delimited for the full extent of South America, with a 2.5’ arc-min to allow the projection of putative distribution areas beyond the known distribution limits, given the relative lack of ecological information available for the species.

The total occurrence dataset was divided into 30 randomized subsets (bootstrap), with 70% and 30% of the occurrence points for training and model testing, respectively. We applied the LPT (Lowest Presence Training [70]) threshold to transform the suitability matrices into presence/absence matrixes, which were then projected for the full extent of South America. We used the True Skilled Statistics (TSS hereon) to evaluate the distributions produced for *A*. *ominicolor*. This evaluation metric ranges from -1 to 1, where values that are negative or close to zero represent models that are not better than a random spatial distribution, while +1 indicates perfect agreement between the species’ known and predicted distribution. Models with TSS values near 0.5 or higher are generally accepted [71].

To assess the potential distribution of *A*. *omnicolor*, we used the mean consensus map of all distributions produced for each climate scenario to determine the climatically stable areas in all scenarios. This approach is considered one of the best methods to acquire the consensus of potential distributions obtained from different algorithms [72].

### ABC

**Fig 2:**
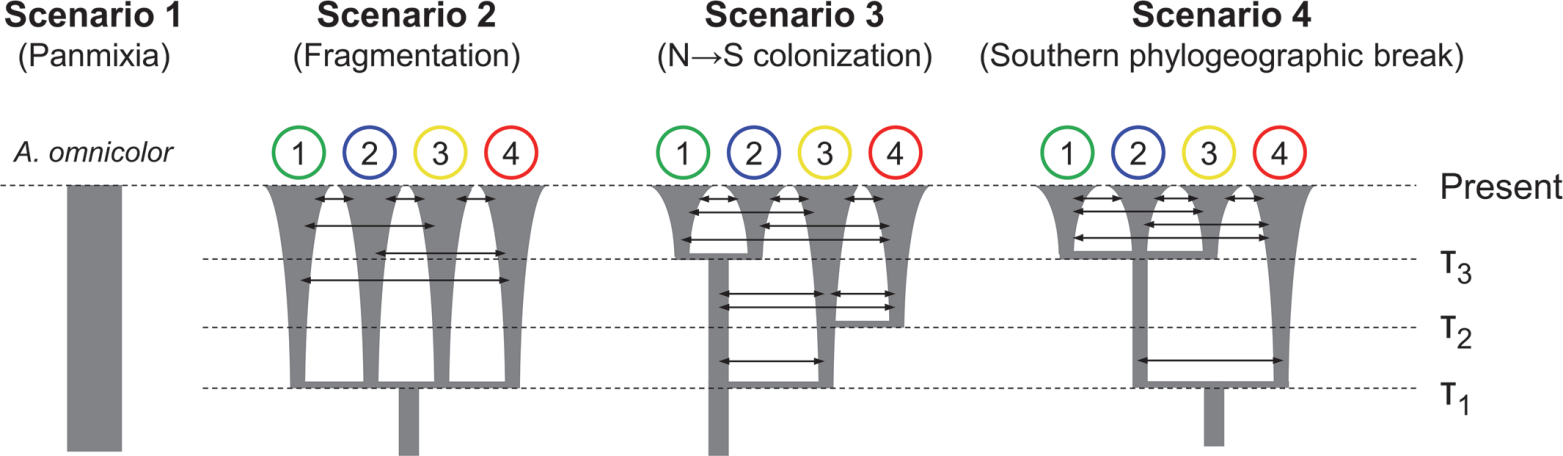
Alternative demographic scenarios for *Araneus omnicolor*. Scenario 1: panmictic population; scenario 2: single fragmentation event partitioning the ancestral population into geographically separated patches; scenario 3: southward colonization; scenario 4: southern phylogeographic break. Only the highest-probability models in each scenario are shown (see results). Numbers above branches indicate geographical regions; arrows and expanding branches represent migration and exponential population growth, respectively. τ = divergence/expansion times (see Table 1).

We used an ABC framework [25–27] to compare four possible demographic scenarios for *A*. *omnicolor* (Fig. 2). Scenario 1 represents a single panmictic population over time, while the others reflect different hypotheses for diversification in the region: scenario 2 reflects a single fragmentation event partitioning the ancestral population into geographically separated patches; scenario 3 illustrates a southward colonization, as several studies have suggested that the southernmost part of Brazil was more unstable climatically during the Quaternary [5,6]; and scenario 4 represents an ancient discontinuity in the southern limit of the Atlantic Rainforest (between 29° and 30° S), a region considered an important phytogeographical disjunction [73] and recognized as a phylogeographic break for some taxa [18,74]. For each scenario, we designed a set of models with different combinations of exponential population growth and migration parameters (total of 14 models, S2 Fig.) and applied a hierarchical procedure similar to that used by Fagundes *et al*. [75], selecting the most likely model within each set and comparing these 4 models to obtain the scenario with the highest probability.

We performed 100 000 data simulations under each model with custom Python scripts in *ms* [76], using the same number and length of loci and sample sizes of empirical data. All parameters were initially drawn from flat prior uniform distributions, and preliminary rejection steps were conducted to restrict the range values (Table 1). The summary statistics based on simulated datasets, including the total nucleotide diversity (π), number of segregating sites (SS), Tajima’s D (D), nucleotide diversity within populations (π_w_) and nucleotide diversity between populations (π_b_), were calculated with a PERL script written by N. Takebayashi (available at: http://raven.iab.alaska.edu/~ntakebay/teaching/programming/coalsim/scripts/msSS.pl).

**Table 1:** Prior distributions of parameters and posterior estimates based on the most probable model.

**Parameter**	**Prior uniform distribution**	**Posterior estimate (95% HPD)**
θ_COI_ (N_e_μ)	0.25–7.0	0.963 (0.696–1.272)
θ_ITS2_ (4N_e_μ)	4 x θ_COI_	3.858 (2.694–5.246)
τ_1_ (generations/4N_e_)^a^	0.01–4.0	-
τ_2_ (generations/4N_e_)	0.001 – τ_1_	-
τ_3_ (generations/4N_e_)	0.001 – τ_2_	-
m (n° migrant copies per generation/n° populations-1)^b^	0.001–15.0	-
α [-(1/τ)*log(N_eτ_/N_e_)]^c^	0.25–0.9	-

θ = theta, τ = divergence/exponential growth times; m = migration rate; α = growth ratio.

^a^τ_1_ = 0.003–10 Ma (absolute time). N_e_ was estimated using the COI substitution rate of 0.0115/My and a value of θ_COI_ previously estimated in DnaSP.

^b^m = 0–5 migrants per generation.

^c^The levels of α tested resulted in 10–75% of population growth.

To assess the most informative summary statistics (i.e., those that most accurately identified the model that best fit the data), we grouped them in vectors and conducted a rejection step using 10 simulations for each model from the prior distribution as pseudo-observed datasets (PODs). The best vector was chosen by its ability to maximize the probability of choosing the true model over the average probability of choosing an incorrect model [Pr(true model)/mean Pr(false models)], following the approach described by Tsai & Carstens [77].

We calculated the posterior probabilities of all competing models and the posterior distributions for the parameters of the most likely model with the R package “abc” [78]. We compared the models, applying two degrees of tolerance (using 0.1% and 1% of the simulations closest to the empirical data) with three methods: simple rejection, multinomial logistic regression [26] and neural network [79]. Finally, we used the simulations under the most probable model to estimate the parameters with the neural network method and a tolerance threshold of 1% of the simulations.

## Results

### Haplotype networks, genetic diversity and population structure

We obtained 668 bp of COI sequences and 274 bp of ITS2 sequences with 37 and 11 polymorphic sites, respectively (Table 2). No stop codons or ambiguous peaks were observed in the electropherograms of the COI sequences, which suggests the absence of nuclear pseudogenes or numts in our mtDNA data. The ITS2 haplotype reconstruction conducted in PHASE resulted in 250 solved sequences (only 10 inferred sequences had a posterior probability lower than 0.6), and no sign of recombination was detected.

**Table 2:** Diversity indices, neutrality tests and results of mismatch distribution analyses for populations and geographical regions.

	**COI**								**ITS2**							
**Population**	**N**	**S**	**H**	**H**_d_ **(s.d.)**	**π (s.d.)**	**D**	**FS**	***r***	**N**	**S**	**H**	**H**_d_ **(s.d.)**	**π (s.d.)**	**D**	**FS**	***r***
Centenário do Sul	13	12	12	0.99 (0.03)	0.005 (0.003)	-0.64	**-9.10**	0.03	28	7	8	0.71 (0.06)	0.006 (0.004)	-0.54	-1.93	0.14
Florestópolis	9	7	5	0.83 (0.10)	0.004 (0.003)	0.25	-0.17	0.05	20	4	4	0.62 (0.07)	0.005 (0.003)	-0.09	0.66	**0.59**
Teodoro Sampaio	15	8	6	0.83 (0.06)	0.004 (0.002)	-0.03	-0.29	0.08	30	7	5	0.66 (0.05)	0.005 (0.003)	-1.25	0.13	0.15
Campinas	11	2	3	0.64 (0.09)	0.001 (0.001)	0.20	-0.02	0.25	20	5	5	0.66 (0.07)	0.005 (0.003)	-0.83	-0.38	0.19
Jundiaí	19	10	5	0.71 (0.08)	0.006 (0.004)	1.66	3.04	0.18	36	2	3	0.54 (0.03)	0.004 (0.003)	1.63	1.85	**0.58**
Ibirama	21	13	12	0.86 (0.07)	0.003 (0.002)	-1.31	**-6.53**	0.04	42	5	4	0.54 (0.04)	0.004 (0.003)	-0.77	1.03	0.75
Gramado	9	3	5	0.81 (0.12)	0.002 (0.001)	0.02	**-2.23**	0.24	18	7	5	0.68 (0.04)	0.007 (0.005)	-0.69	0.35	0.29
Sapiranga	33	11	10	0.72 (0.08)	0.002 (0.001)	**-1.56**	**-4.77**	0.05	56	9	9	0.69 (0.04)	0.006 (0.004)	-0.80	-1.61	**0.35**
Region 1	37	17	16	0.91 (0.02)	0.004 (0.002)	-1.02	**-7.22**	0.03	78	7	9	0.61 (0.03)	0.005 (0.004)	-0.47	-1.68	**0.40**
Region 2	30	11	6	0.77 (0.04)	0.007 (0.004)	1.93	3.45	0.08	56	5	5	0.54 (0.03)	0.004 (0.003)	-0.67	1.27	**0.57**
Region 3	21	13	12	0.86 (0.07)	0.003 (0.002)	-1.31	**-6.53**	0.04	42	5	4	0.54 (0.04)	0.004 (0.003)	-0.77	1.03	0.75
Region 4	42	13	14	0.74 (0.07)	0.002 (0.001)	**-1.73**	**-10.00**	0.06	74	11	10	0.66 (0.04)	0.006 (0.004)	-1.05	-1.85	**0.43**
Total	130	37	42	0.92 (0.01)	0.005 (0.003)	**-1.56**	**-26.22**	0.02	250	12	17	0.63 (0.02)	0.005 (0.003)	-1.14	-7.09	**0.35**

N = n° of sequences; S = n° of polymorphic sites; H = n° of haplotypes; H_d_ = haplotype diversity; π = nucleotide diversity; s.d. = standard deviation; D = Tajima’s D; FS = Fu’s FS; *r* = Harpending’s raggedness index. In bold, the statistical significant values (p<0.05).

The haplotype and nucleotide diversities observed with both markers showed low variation among populations and geographical regions, although the number of COI haplotypes was higher than for ITS2 (42 and 17, respectively) (Fig. 1B, Fig. 1C, Table 2). Most COI haplotypes were exclusive: 7 were found in more than one population and only 2 were observed in at least 3 geographical regions (no haplotype was found in all regions, Fig. 1B). These two more widely distributed haplotypes were also the most common, linking several less-frequent haplotypes in a star-shaped network (Fig. 1B). In the ITS2 network, the two predominant haplotypes were found in all geographical regions, and most heterozygous individuals contained these two sequences (Fig. 1C).

The genetic distances between populations were also low (S2 Table). The mean number of COI nucleotide differences ranged from 0.3 to 3.16, and the differences were not significantly correlated with geographical distance (r = 0.238, p = 0.081); for ITS2 sequences, all distance values were non-significant.

The COI pairwise Φ_ST_ values ranged from 0.065 to 0.719 (Table 3) and showed a significant correlation with geographical distance (r = 0.505, p = 0.005); the AMOVA indicated that only 10.6% of this variation was observed among geographical regions (S3 Table). For the ITS2 dataset, all Φ_ST_ values were non-significant and the AMOVA showed that 99.9% of the variance was intrapopulational, indicating no population structure (Table 3, S3 Table).

**Table 3:** Population pairwise Φ_ST_ for COI (below diagonal) and ITS2 (above diagonal) datasets.

	**Centenário do Sul**	**Florestópolis**	**Teodoro Sampaio**	**Campinas**	**Jundiaí**	**Ibirama**	**Gramado**	**Sapiranga**
**Centenário do Sul**	-	-0.022	-0.024	0.001	-0.020	0.047	-0.039	-0.010
**Florestópolis**	-0.020	-	-0.004	-0.028	-0.011	0.015	-0.036	-0.023
**Teodoro Sampaio**	-0.014	0.069	-	-0.016	-0.001	0.030	-0.030	-0.015
**Campinas**	**0.183**	**0.312**	**0.262**	-	0.031	-0.026	-0.014	-0.010
**Jundiaí**	**0.377**	**0.427**	**0.362**	**0.511**	-	-0.016	0.009	0.011
**Ibirama**	**0.065**	**0.113**	0.070	**0.138**	**0.433**	-	0.031	0.029
**Gramado**	**0.232**	**0.339**	**0.255**	**0.719**	**0.435**	**0.366**	-	-0.027
**Sapiranga**	**0.251**	**0.382**	**0.233**	**0.602**	**0.505**	**0.340**	0.030	-

In bold, the significant values (p<0.05).

### Phylogenetic inferences and divergence times

**Fig 3:**
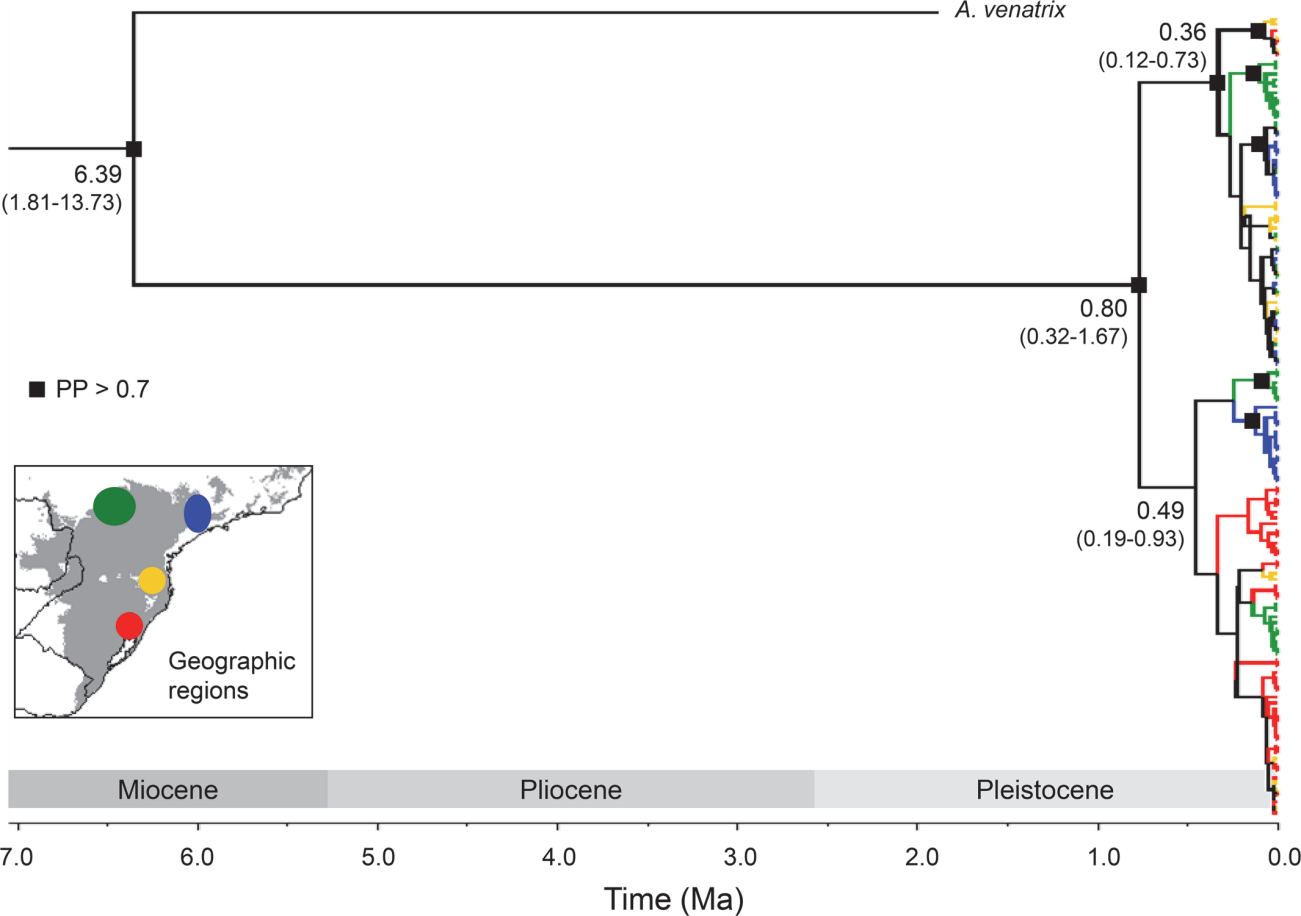
Bayesian phylogenetic inference for COI sequences. The divergence times of the main nodes are shown, with 95% HPD in parentheses. Black squares represent nodes with posterior probability > 0.7. Branch colors correspond to the geographical regions studied (map in detail).

The COI and ITS2 phylogenetic inferences recovered different topologies and many low posterior-probability nodes (Fig. 3, S3 Fig.). In the time-calibrated COI tree, the divergence from the outgroup (*A*. *venatrix*) was estimated at ca. 6.39 Ma (95% HPD = 1.81–13.73 Ma), but most of the species diversification occurred in the last 0.5 Ma (Fig. 3). The COI sequences were split into two clades with a weak geographical association. However, this subdivision was not considered, because of the overlapping confidence intervals of the node ages and low statistical support in one of the clades (Fig. 3).

### Demographic analyses

**Fig 4:**
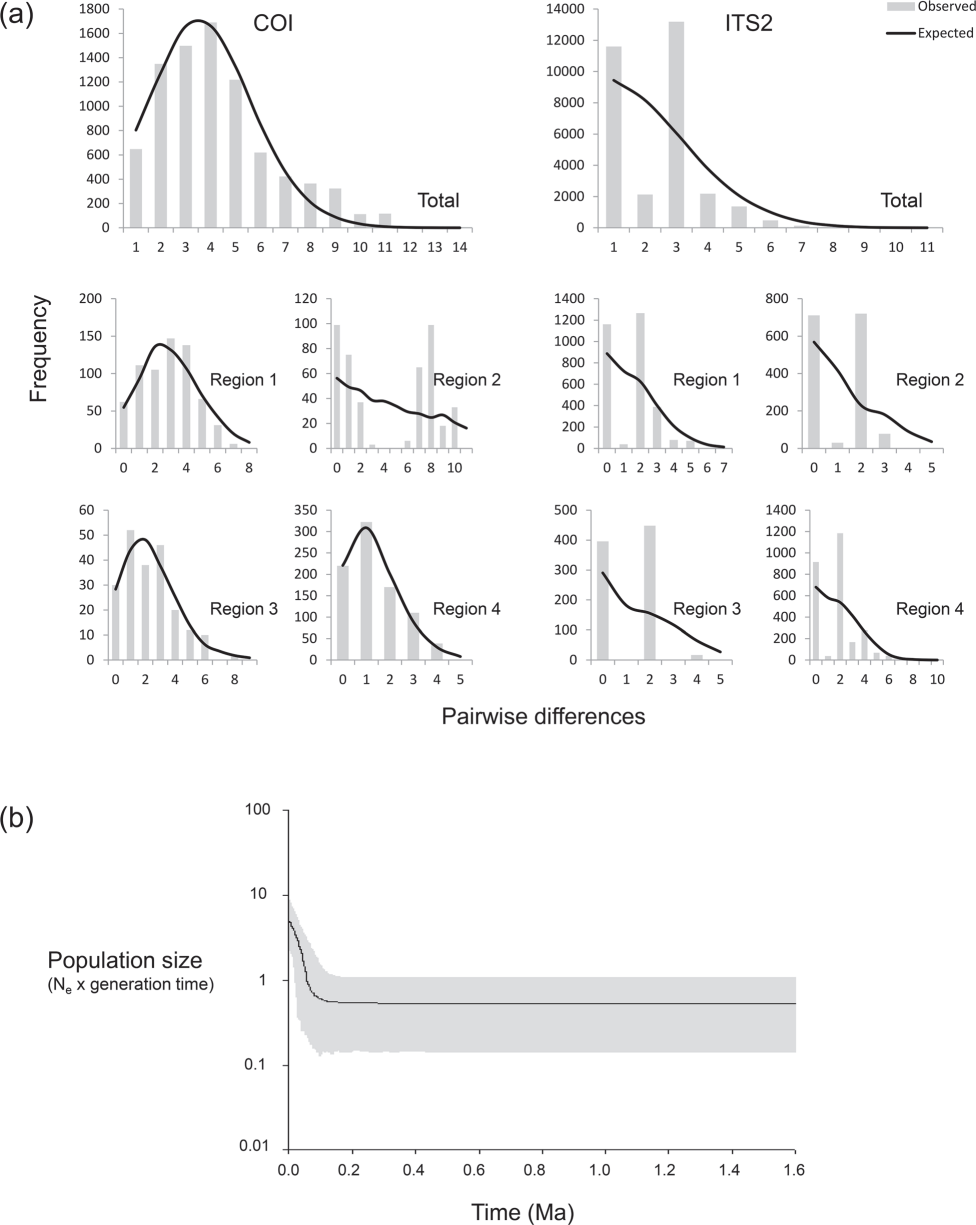
Results of demographic analyses. (a) Results of mismatch distribution analyses for COI (left) and ITS2 (right) total datasets and for each geographical region separately. (b) Demographic expansion detected by multilocus EBSP, with the 95% HPD interval shown in gray.

Neutrality tests detected demographic expansion in the COI sequences for several populations (Table 2). When geographical regions were considered, 3 of the 4 groups showed significant negative values for at least one test (except for region 2), and both Tajima’s D and Fu’s Fs indicated expansion in the total COI dataset (Table 2). Mismatch distribution analyses generated similar results, as shown by the unimodal curves of COI pairwise difference frequencies and the non-significant raggedness indices (Fig. 4A, Table 2). For the nuclear sequences, neutrality tests and mismatch distribution analyses detected no evidence of demographic events (Fig. 4A, Table 2). The multilocus EBSP revealed a large demographic expansion in *A*. *omnicolor* ca. 0.1 Ma (considering that the species has one generation per year, Fig. 4B).

### Species distribution and paleoclimate modeling

**Fig 5:**
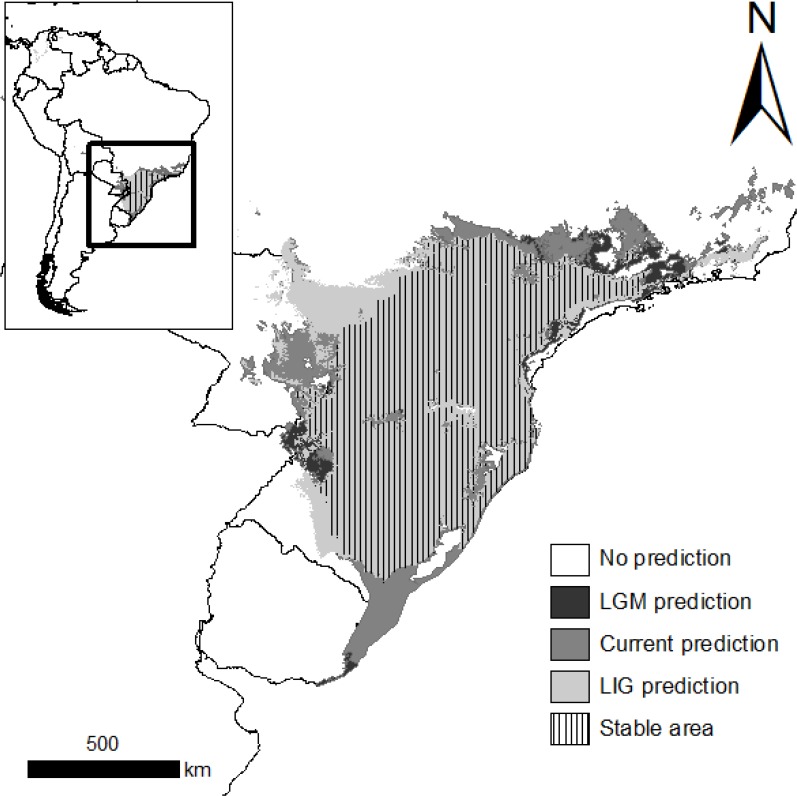
Modeled distributions of *Araneus omnicolor*. The distributions in the current and paleoclimate [21 Kya (LGM) and 120 Kya (LIG)] scenarios are represented. The hatched area represents the stable occurrence region during all periods.

Our species distribution models showed reliable predictions with all algorithms, as evidenced by the high TSS values (GARP = 0.909 ± 0.017; SVM = 0.813 ± 0.066; Maxent = 0.842 ± 0.074). The models exhibited similar distribution patterns in all three climate scenarios, indicating niche stability in *A*. *omnicolor* during the last 120 Kya (hatched area in Fig. 5).

### ABC

We used the summary statistics vector comprising only the nucleotide diversity indices (π, π_w_ and π_b_), which was the most informative for our dataset. In the first approach to the model selection (within scenarios), we observed models with consistent highest probabilities in scenarios 1 and 3 with all methods applied, but found different results for scenarios 2 and 4 among the different methods (S4 Table). Because simple rejection was the only method that provided congruent results with both tolerance thresholds in all scenarios, we used only this method with a threshold of 0.1% for the model selection.

In scenario 1 (panmixia), the probability of a stable population model (no population growth) was slightly higher, although the probabilities between models were not significantly different (Table 4). In all population differentiation scenarios (2, 3 and 4), the models including migration had the highest probabilities (Table 4). In the final model comparison (among scenarios), the model of panmixia with constant population size showed the highest probability (0.63, Table 4).

**Table 4:** Model selection within and among scenarios based on rejection method.

		**Posterior model probability (rejection method)**	
**Scenario**	**Model**	**Within scenarios**	**Among scenarios**
1 (Panmixia)	**1**	**0.5311**	**0.6267**
	2 (N_e_ expansion)	0.4689	
2 (Fragmentation)	3	0.1259	
	4 (migration)	0.3925	
	5 (N_e_ expansion)	0.0729	
	**6 (migration, N**_e_ **expansion)**	**0.4087**	0.0648
3 (N→S colonization)	7	0.1195	
	8 (migration)	0.3707	
	9 (N_e_ expansion)	0.1075	
	**10 (migration, N**_e_ **expansion)**	**0.4023**	0.1794
4 (Southern phylogeographic break)	11	0.1054	
	12 (migration)	0.3605	
	13 (N_e_ expansion)	0.1479	
	**14 (migration, N**_e_ **expansion)**	**0.3863**	0.1291

The highest-probability model in each scenario is shown in bold.

Estimation of parameters using the neural network method was highly informative compared to both the prior distribution and the regular rejection approach (S4 Fig.). The values of θ_COI_ and θ_ITS2_ were close to the lower limits of our prior distribution: 0.96 (95% HPD 0.7–1.27) and 3.86 (95% HPD 2.7–5.25), respectively (Table 1).

## Discussion

### Genetic diversity, population structure and incongruence between mtDNA and nrDNA patterns

*A*. *omnicolor* exhibited high haplotype diversity, but low nucleotide variation in the haplotypes (Fig. 1, Table 2, S2 Table). The COI and ITS2 total nucleotide diversities were estimated as 0.5%, whereas the mean intraspecific values for spiders are 2.15% and 1%, respectively [80,81].

The genetic diversity indices showed slight variation among geographical regions (Table 2), which lowers the possibility of inferring a likely ancestral origin or possible refugia in the past (i.e., areas with higher variability). Although paleoecological studies have suggested that the southernmost region of Brazil underwent more drastic climate and floristic changes during the Quaternary [5,6], our results do not support a recent southward colonization by this species, as its variability did not decrease in higher latitudes.

A significant population structure was detected by the mitochondrial marker, but not by the nrDNA (Table 3). Differences between COI and ITS2 were also observed in the haplotype networks (high number of exclusive COI haplotypes, while the more frequent ITS2 haplotypes were widespread, Fig. 1) and demographic analyses (expansion detected only by COI, Table 2, Fig. 4A). This incongruence is commonly observed in several taxa, which tend to exhibit a more evident phylogeographic structure with uniparental inherited markers [3,82]. There are several possible reasons for this pattern, such as incomplete sorting of nuclear lineages (given the higher evolutionary rate and lower N_e_ in mtDNA), nuclear introgression, mitochondrial selection or demographic asymmetries, e.g., different migration behaviors of males and females [83,84]. Male-biased dispersal is frequent in spider species in which the adult females are larger and more sedentary than the males, which actively seek for females [85,86]. Therefore, our findings suggest that females of *A*. *omnicolor* might have limited dispersal (corroborated by the evidence of isolation by distance in the mtDNA results) and the genetic connectivity in the species is mainly caused by long-distance migration of the males.

### Phylogenetic inferences and divergence time

The COI Bayesian inference indicated that *A*. *omnicolor* originated in the Late Miocene (6.39 Ma, 95% HPD = 1.81–13.73 Ma), but most of the species’ diversification occurred much more recently (Late Pleistocene, Fig. 3). The low nucleotide variance detected among sequences supports this recency of diversity in the species (Table 2), although the application of a COI substitution rate proposed for insects requires a cautious interpretation of the divergence times. Several previous studies with Neotropical taxa have reported intense lineage radiation in the same period, a pattern generally attributed to the effects of Pleistocene climate oscillations [3]. Despite the stability of the distribution of *A*. *omnicolor* during Late Pleistocene (Fig. 5), we cannot rule out the possibility that earlier climate fluctuations in this period are linked to the pattern observed (see discussion below).

### Demographic analyses

Our analyses indicated a demographic expansion beginning around 100 Kya (Fig. 4B). The result suggests that Pleistocene climate oscillations have not affected *A*. *omnicolor* population growth since the LIG (~120 Kya), even though several other South American subtropical taxa exhibit signs of population bottlenecks during this period [17,19,87,88]. This different pattern might be explained by the broader ecological resilience of *A*. *omnicolor*, which is found in a variety of biomes, compared with most of the species so far studied in this region, which are restricted to the Atlantic Rainforest. As a species’ response to climate changes depends strongly on its ecological and environmental tolerances [89], the effects of Pleistocene oscillations are, indeed, expected to be more pronounced in organisms that are narrowly associated with a specific phytophysiognomy. Similar results were reported by Batalha-Filho *et al*. for the passerine bird *Basileuterus leucoblepharus*, which occurs in the southern Atlantic Rainforest and also in other subtropical formations [16]. However, further conclusions should be formed only with care, since the estimate of a demographic expansion of *A*. *omnicolor* was based on node calibration under a standard COI mutation rate.

### Species distribution and paleoclimate modeling

The species distribution models and paleoclimate reconstructions indicated that the area potentially occupied by *A*. *omnicolor* remained stable during the last 120 Kyr (Fig. 5), contradicting the intense landscape modification attributed to this region during the Late Pleistocene [5]. This niche stability agrees with the genetic diversity and nrDNA structure observed, since the lack of fragmentation of the distribution area might have allowed efficient gene flow during this entire period.

The influence of Quaternary climate fluctuations on the distribution of South American subtropical fauna is still poorly understood, and, as for phylogeographic studies, paleoclimate modeling has mostly focused on taxa that are endemic to the Atlantic Rainforest. Despite the drastic fragmentation of the southern portion of this biome detected by paleoclimate models [19,90], Carnaval *et al*. showed that several groups have exhibited niche stability since the LIG, suggesting that the ‘forest stability’ (i.e., the persistence of a particular forest-type environment) in this region is higher than previously predicted [91].

As suggested for the demographic patterns, the occurrence of *A*. *omnicolor* through several biomes (not restricted to the Atlantic Rainforest) could be another important factor for the constancy of the species’ niche. In a study with Neotropical orchid bees, for example, the authors demonstrated that the species with wider physiological tolerances to climate conditions underwent less-drastic niche reductions during LGM [82]. Therefore, our niche modeling—as well as the demographic analyses—highlights the influence of specific ecological tolerances on organisms’ responses to climate changes.

### ABC

A panmictic population with constant size is the demographic scenario that best explained the current phylogeographic patterns of *A*. *omnicolor* (Fig. 2, Table 4). The role of connectivity for the species was also evidenced by comparisons within the remaining scenarios, as all the highest probability models included migration (Table 4). These results agree with the niche stability detected by our paleoclimate modeling, indicating that the species was not significantly impacted by LGM climate peaks.

Although the selected model predicts N_e_ stability (no population growth), we cannot dismiss the importance of demographic expansion in *A*. *omnicolor*, as the probability of the exponential growth model was slightly lower for the panmictic scenario, and all chosen models in subdivision scenarios also included expansion (Table 4). However, the time of this event is unclear, as the parameter estimation was based on the model without exponential growth. Thus, our ABC results are consistent with a possible demographic expansion occurring before the Late Pleistocene or driven by factors other than climate, as suggested by the genetic analyses.

## Conclusions

Our study indicated that the subtropical spider *A*. *omnicolor* experienced no niche reduction or demographic declines during the Late Pleistocene. The high dispersal ability of this species, together with its wide environmental and ecological tolerances, might have provided the niche stability observed. Further, the recent diversification and demographic expansion detected in our results suggest that other factors than LIG and LGM climate oscillations may have affected the evolutionary history of this species. These patterns differ substantially from those seen for many other groups, and emphasize the importance of extending phylogeographic investigations to taxa that are less-often studied, such as invertebrates. Finally, more studies with species that are widely distributed in different biomes are essential for a complete understanding of the effects of Quaternary climate changes on Neotropical biodiversity.

## Supporting Information

S1 Fig Occurrence records of *Araneus omnicolor*. Compilation of all occurrence records for the species, including points from the literature and zoological databases (red circles) and new records obtained in this study (blue circles).
(TIF)

S2 Fig Alternative demographic models compared in ABC analysis. Scenario 1: panmictic population; scenario 2: a single fragmentation event partitioning the ancestral population into geographically separated patches; scenario 3: southward colonization; scenario 4: southern phylogeographic break (for more details, see text). Variations within scenarios include migration (represented by arrows), exponential population growth (represented by expansion in branches) or both. τ = divergence/expansion times (see Table 1).
(TIF)

S3 Fig Bayesian phylogenetic inference for ITS2 sequences. Branch lengths are shown in number of substitutions, and colors correspond to the geographical regions studied (map in detail). Black squares represent nodes with posterior probability > 0.7.
(TIF)

S4 Fig Results of parameter estimation. (a) θ_COI_ and (b) θ_ITS2_ estimation plots resulting from the R package ‘abc’.
(TIF)

S1 Table Bioclimate variables used in the distribution modeling. Relative contributions of environmental variables to the models, using the Jackknife procedure. In bold, the five variables selected for the analyses.
(DOCX)

S2 Table Genetic distances between populations. Corrected number of COI (below diagonal) and ITS2 (above diagonal) nucleotide substitutions between populations (*D*_A_ [41]). In bold, the significant values (p<0.05).
(DOCX)

S3 Table Analyses of molecular variance. AMOVA results based on COI and ITS2 sequences (d.f. = degrees of freedom; *p<0.001; **p<0.01).
(DOCX)

S4 Table Model selection within scenarios with different methods (simple rejection, logistic regression and neural network). The highest-probability model in each scenario is shown in bold; the models selected with the rejection model (used in the comparison among scenarios) are highlighted in gray. T = simulation threshold.
(DOCX)
