# Supplementary figures and images for: Pleistocene Niche Stability and Lineage Diversification in the Subtropical Spider Araneus omnicolor (Araneidae)
Source: PLoS One. 2015 Apr 9;10(4):e0121543. doi: 10.1371/journal.pone.0121543 (PMC4391720; doi:10.1371/journal.pone.0121543)

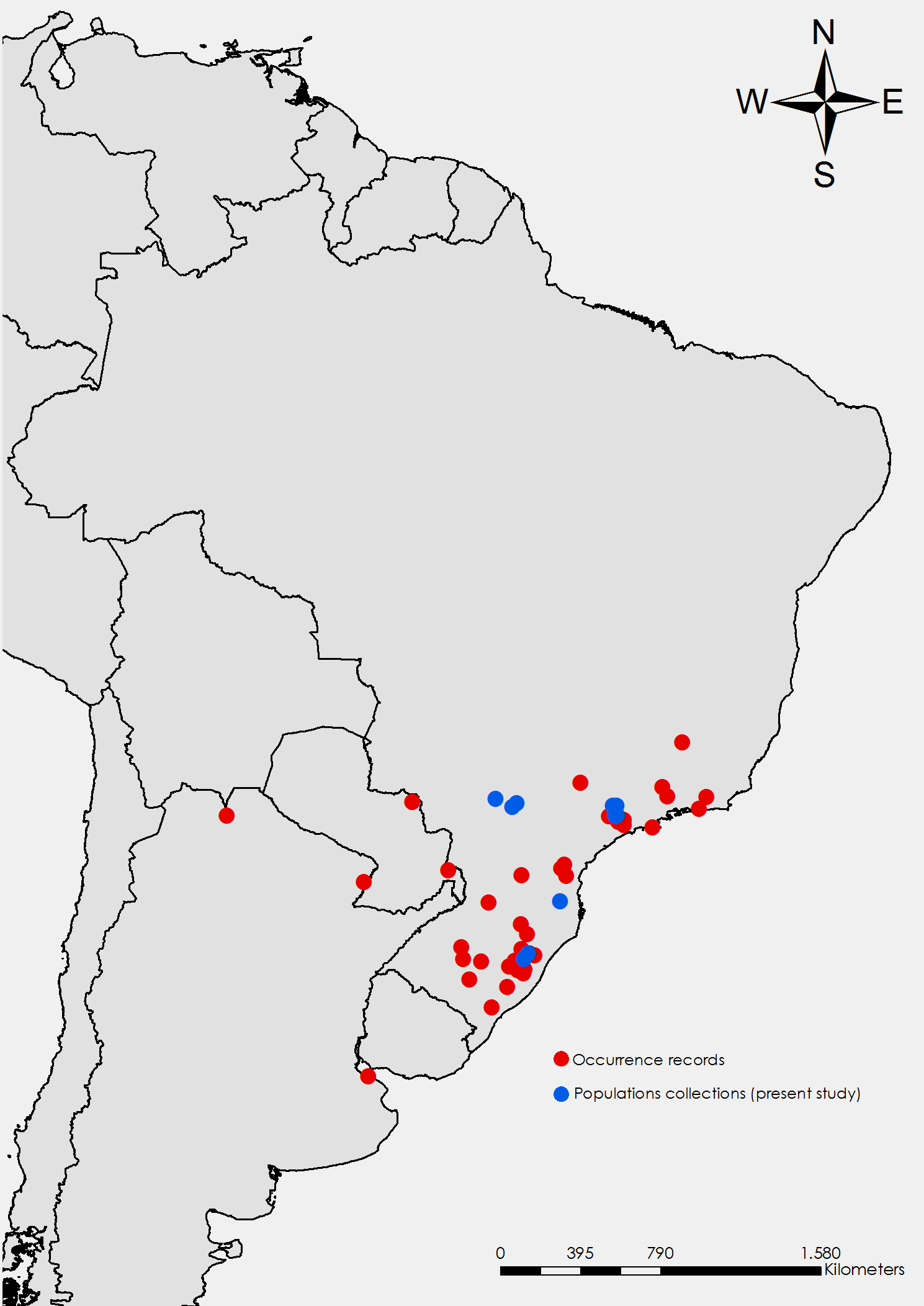

Supplement: S1 Fig — Compilation of all occurrence records for the species, including points from the literature and zoological databases (red circles) and new records obtained in this study (blue circles). (TIF) [file pone.0121543.s001.tif]

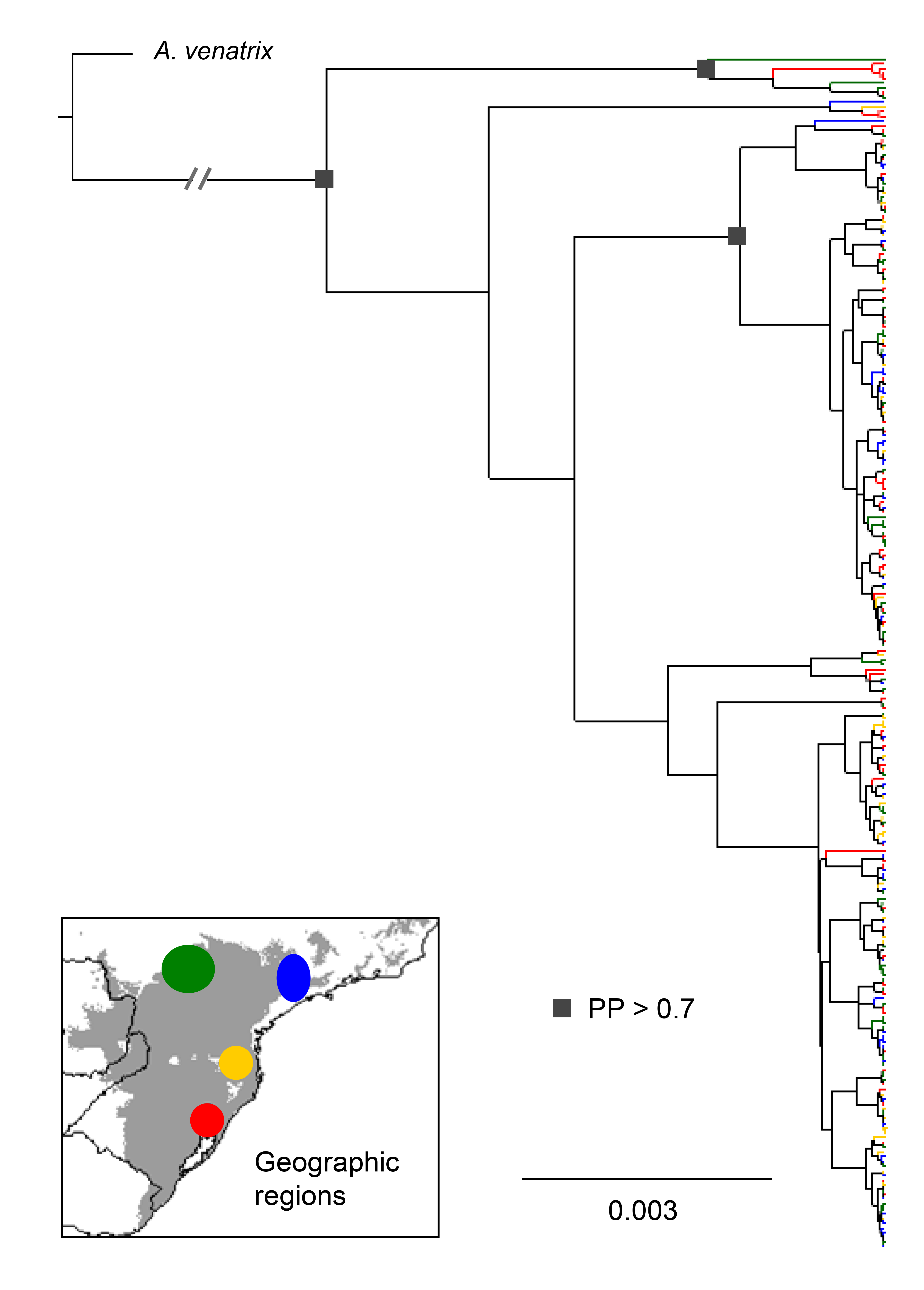

Supplement: S3 Fig — Branch lengths are shown in number of substitutions, and colors correspond to the geographical regions studied (map in detail). Black squares represent nodes with posterior probability > 0.7. (TIF) [file pone.0121543.s003.tif]

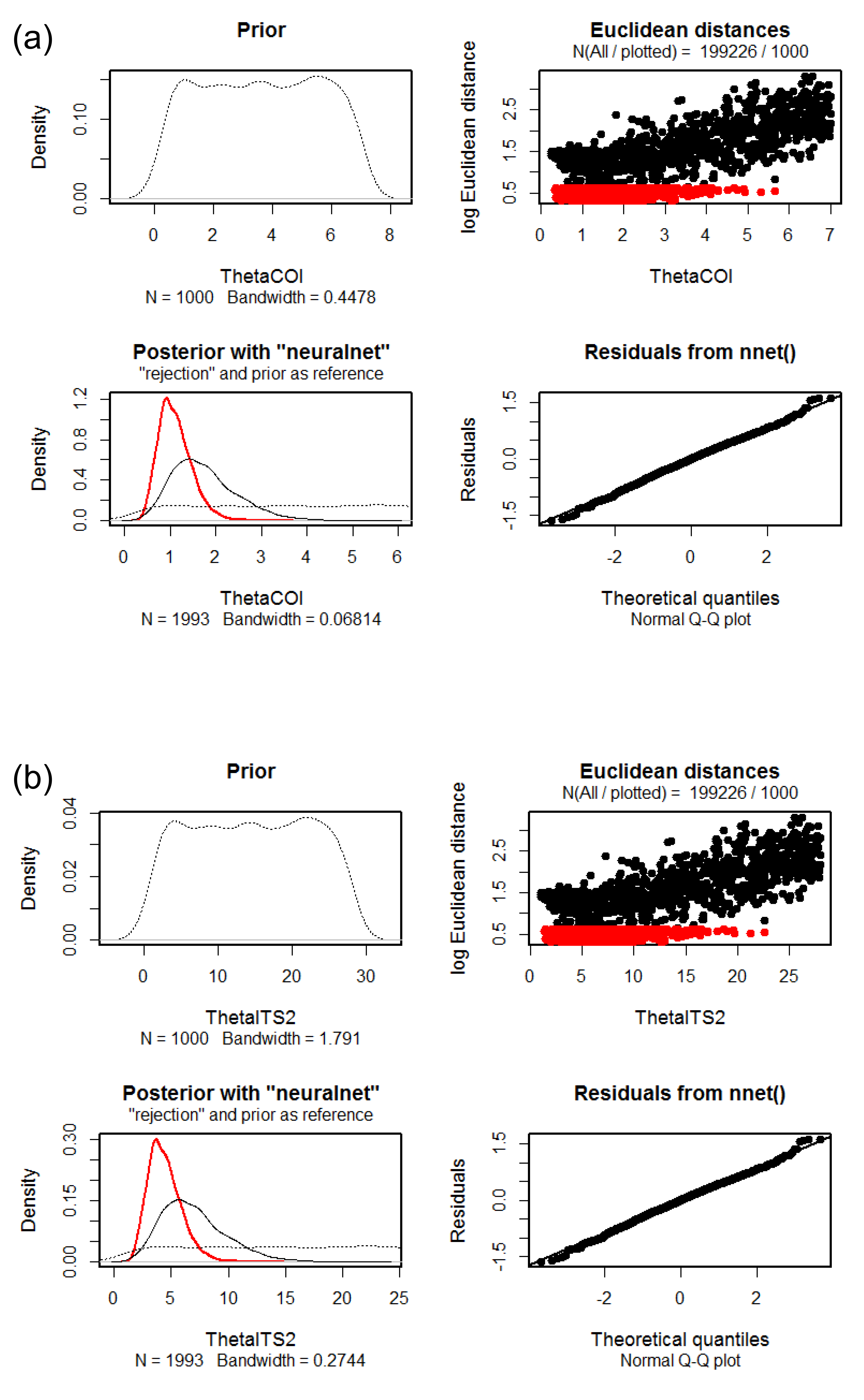

Supplement: S4 Fig — (a) θCOI and (b) θITS2 estimation plots resulting from the R package ‘abc’. (TIF) [file pone.0121543.s004.tif]
