# Supplementary material for: Pleistocene Niche Stability and Lineage Diversification in the Subtropical Spider Araneus omnicolor (Araneidae)
Source: PLoS One. 2015 Apr 9;10(4):e0121543. doi: 10.1371/journal.pone.0121543 (PMC4391720; doi:10.1371/journal.pone.0121543)
Supplement: S1 Table — Relative contributions of environmental variables to the models, using the Jackknife procedure. In bold, the five variables selected for the analyses. (DOCX) [file pone.0121543.s005.docx]

| **Variable** | **Percent contribution** | **Permutation importance** |
| --- | --- | --- |
| **Precipitation of Driest Month** | **30.3** | **3.5** |
| **Max Temperature of Warmest Month** | **15** | **0.5** |
| **Precipitation of Warmest Quarter** | **15** | **30.3** |
| Mean Diurnal Range* | 10.2 | 6.5 |
| **Mean Temperature of Coldest Quarter** | **9.2** | **8.6** |
| **Temperature Seasonality** | **8.1** | **19** |
| Temperature Annual Range | 5.6 | 23.2 |
| Precipitation Seasonality | 2.2 | 2.5 |
| Min Temperature of Coldest Month | 2 | 1.8 |
| Mean Temperature of Coldest Quarter | 1.3 | 0.6 |
| Annual Mean Temperature | 0.5 | 1.6 |
| Mean Temperature of Wettest Quarter | 0.2 | 0.1 |
| Mean Temperature of Driest Quarter | 0.1 | 0.6 |
| Annual Precipitation | 0.1 | 0.4 |
| Isothermality | 0.1 | 0.6 |
| Precipitation of Driest Quarter | 0 | 0 |
| Precipitation of Wettest Quarter | 0 | 0 |
| Precipitation of Wettest Month | 0 | 0 |
| Mean Temperature of Warmest Quarter | 0 | 0 |

*We discarded the ‘Mean Diurnal Range’ variable and used ‘Temperature Seasonality’ instead because, although their similar relative contributions (10.2% and 8.1%, respectively), the permutation importance of the latter was considerably higher (19).
