# Supplementary material for: Pleistocene Niche Stability and Lineage Diversification in the Subtropical Spider Araneus omnicolor (Araneidae)
Source: PLoS One. 2015 Apr 9;10(4):e0121543. doi: 10.1371/journal.pone.0121543 (PMC4391720; doi:10.1371/journal.pone.0121543)
Supplement: S2 Table — Corrected number of COI (below diagonal) and ITS2 (above diagonal) nucleotide substitutions between populations (D A [41]). In bold, the significant values (p<0.05). (DOCX) [file pone.0121543.s006.docx]

|  | Centenário do Sul | Florestópolis | Teodoro Sampaio | Campinas | Jundiaí | Ibirama | Gramado | Sapiranga |
| --- | --- | --- | --- | --- | --- | --- | --- | --- |
| Centenário do Sul | - | -0.030 | -0.037 | 0.003 | -0.024 | 0.064 | -0.069 | -0.017 |
| Florestópolis | -0.049 | - | -0.046 | -0.036 | -0.015 | 0.015 | -0.057 | -0.030 |
| Teodoro Sampaio | -0.042 | 0.183 | - | -0.021 | -0.002 | 0.037 | -0.051 | -0.021 |
| Campinas | **0.491** | **0.710** | **0.635** | - | -0.037 | -0.033 | -0.024 | -0.010 |
| Jundiaí | **2.321** | **2.832** | **1.962** | **3.159** | - | -0.017 | -0.001 | 0.020 |
| Ibirama | 0.169 | **0.295** | 0.176 | **0.320** | **2.442** | - | 0.031 | 0.045 |
| Gramado | **0.765** | **0.985** | **0.699** | **2.293** | **2.591** | **1.170** | - | 0.049 |
| Sapiranga | **0.588** | **0.960** | **0.495** | **1.860** | **2.406** | **0.881** | 0.052 | - |
