# Supplementary material for: Pleistocene Niche Stability and Lineage Diversification in the Subtropical Spider Araneus omnicolor (Araneidae)
Source: PLoS One. 2015 Apr 9;10(4):e0121543. doi: 10.1371/journal.pone.0121543 (PMC4391720; doi:10.1371/journal.pone.0121543)
Supplement: S3 Table — AMOVA results based on COI and ITS2 sequences (d.f. = degrees of freedom; *p<0.001; **p<0.01). (DOCX) [file pone.0121543.s007.docx]

| Source of variation | d.f. | Sum of squares | Variance components | Variation percentage | Fixation indices |
| --- | --- | --- | --- | --- | --- |
| *COI* |  |  |  |  |  |
| Among regions | 3 | 46.148 | 0.185 | 10.60 | F_CT_ = 0.106 |
| Among populations within regions | 4 | 27.058 | 0.431 | 24.64 | F_ST_ = 0.352* |
| Within populations | 122 | 138.256 | 1.133 | 64.76 | F_SC_ = 0.276* |
| *ITS2* |  |  |  |  |  |
| Among regions | 3 | 3.682 | 0.020 | 2.78 | F_CT_ = 0.028** |
| Among populations within regions | 4 | 0.869 | -0.019 | -2.66 | F_ST_ = 0.001 |
| Within populations | 242 | 171.349 | 0.708 | 99.88 | F_SC_ = -0.027 |
