# Supplementary material for: Pleistocene Niche Stability and Lineage Diversification in the Subtropical Spider Araneus omnicolor (Araneidae)
Source: PLoS One. 2015 Apr 9;10(4):e0121543. doi: 10.1371/journal.pone.0121543 (PMC4391720; doi:10.1371/journal.pone.0121543)
Supplement: S4 Table — The highest-probability model in each scenario is shown in bold; the models selected with the rejection model (used in the comparison among scenarios) are highlighted in gray. T = simulation threshold. (DOCX) [file pone.0121543.s008.docx]

|  | | Posterior model probability | | |
| --- | --- | --- | --- | --- |
| Scenario | Model |  |  |  |
|  |  | Rejection method | Logistic regression method | Neural network method |
|  |  | (T = 0.001/0.01) | (T = 0.001/0.01) | (T = 0.001/0.01) |
| 1 (Panmixia) | **1** | **0.5311** / **0.5101** | **0.9966** / **0.787** | **0.8455** / **0.6668** |
|  | 2 (N_e_ expansion) | 0.4689 / 0.4899 | 0.0034 / 0.213 | 0.1545 / 0.3332 |
| 2 (Fragmentation) | 3 | 0.1259 / 0.0985 | 0.045 / **0.3263** | 0.0104 / **0.3013** |
|  | 4 (migration) | 0.3925 / 0.3703 | **0.431** / 0.2358 | 0.3261 / 0.2248 |
|  | 5 (N_e_ expansion) | 0.0729 / 0.1151 | 0.1427 / 0.209 | 0.2617 / 0.279 |
|  | **6 (migration, N_e_ expansion)** | **0.4087** / **0.4161** | 0.3814 / 0.2289 | **0.4018** / 0.1948 |
| 3 (N→S colonization) | 7 | 0.1195 / 0.1075 | 0.0803 / 0.148 | 0.1167 / 0.0469 |
|  | 8 (migration) | 0.3707 / 0.3684 | 0.2086 / 0.3258 | 0.1081 / 0.3548 |
|  | 9 (N_e_ expansion) | 0.1075 / 0.115 | 0.0613 / 0.1323 | 0.01 / 0.0243 |
|  | **10** **(migration, N_e_ expansion)** | **0.4023** / **0.409** | **0.6497** / **0.394** | **0.7651** / **0.5741** |
| 4 (Southern phylogeographic break) | 11 | 0.1054 / 0.1124 | 0.154 / 0.258 | **0.467** / **0.3716** |
|  | 12 (migration) | 0.3605 / 0.3625 | **0.4091** / **0.2802** | 0.2654 / 0.1791 |
|  | 13 (N_e_ expansion) | 0.1479 / 0.1321 | 0.3019 / 0.2659 | 0.1451 / 0.3194 |
|  | **14** **(migration, N_e_ expansion)** | **0.3863** / **0.393** | 0.1351 / 0.1959 | 0.1225 / 0.13 |
